# Micromagnetic Simulation of *L*1_0_-FePt-Based Transition Jitter of Heat-Assisted Magnetic Recording at Ultrahigh Areal Density

**DOI:** 10.3390/mi13101559

**Published:** 2022-09-20

**Authors:** Chavakon Jongjaihan, Arkom Kaewrawang

**Affiliations:** Magnetic Materials and Applications Research Laboratory, Department of Electrical Engineering, Faculty of Engineering, Khon Kaen University, Khon Kaen 40002, Thailand

**Keywords:** transition jitter, heat-assisted magnetic recording, magnetic footprints

## Abstract

The areal density of hard disk drives increases every year. Increasing the areal density has limitations. Therefore, heat-assisted magnetic recording (HAMR) technology has been the candidate for increasing the areal density. At ultrahigh areal density, the main problem of the magnetic recording process is noise. Transition jitter is noise that affects the read-back signal. Hence, the performance of the magnetic recording process depends on the transition jitter. In this paper, the transition jitter of *L*1_0_-FePt-based HAMR technology was simulated at the ultrahigh areal density. The micromagnetic simulation was used in the magnetic recording process. The average grain size was 5.1 nm, and the standard deviation was 0.08 nm. The recording simulation format was five tracks in a medium. It was found that a bit length of 9 nm with a track width of 16.5 nm at the areal density of 4.1 Tb/in^2^ had the lowest transition jitter average of 1.547 nm. In addition, the transition jitter average decreased when increasing the areal density from 4.1 to 8.9 Tb/in^2^. It was found that the lowest transition jitter average was 1.270 nm at an 8 nm track width and a 9 nm bit length, which achieved an ultrahigh areal density of 8.9 Tb/in^2^.

## 1. Introduction

The trend of the areal density (AD) in magnetic recording technology increases every year [1]. It can increase with increased bit density, increased track density, and reduced grain size [2,3]. However, the effects of reducing grain size decrease the thermal stability, which causes superparamagnetic effects. The thermal stability can increase by increasing the magnetocrystalline anisotropy constant, *K*_u_. Magnetic materials are modified to accommodate increasing the areal density for the magnetic recording technology. The *L*1_0_-FePt medium is currently selected as a candidate because of the suitability of the magnetic properties [4,5,6,7,8]. The high *K*_u_, the high saturation magnetization, *M*_s_, and the low curie temperature, *T*_c_, are the magnetic properties of *L*1_0_-FePt that have been optimized for the new technology of hard disks, such as MAMR [9] and HAMR [4,10,11,12,13,14,15].

Heat-assisted magnetic recording (HAMR) technology is chosen to assist magnetic recording at high AD [8,16,17] due to the high *K*_u_ of the magnetic materials. In addition, one of the main problems that occurs in the magnetic recording process is the noise that decreases the signal-to-noise ratio (SNR) or increases the error of the read-back signal. The noise mainly consists of DC noise and jitter noise; consequently, they cause irregular amplitude and make the read-back signal transition less sharp, respectively. The correlation of the noise and the transition jitter, $\sigma_{jitter}$, is strong [18,19,20]. Therefore, the performance of the magnetic recording process is indicated by the $\sigma_{jitter}$. The main causes of the $\sigma_{jitter}$ are the grain size, grain size distribution, grain shape, read width, heat spot geometry, and thermal gradient [10,11,12,19,21,22].

Many publications have simulated magnetic recording to achieve high areal density and high performance. The transition jitter has been used to indicate the performance of the magnetic recording process [12,13,14,15,22]. Valcu and Yeh [22] have improved Voronoi-pattern media for very close to the microtrack model prediction. The transition jitter is used to indicate the efficiency of Voronoi-pattern media and that the detection positions are the zero crossings. It was found that the read width is inversely proportional to the jitter. Niranjan and Victora [12] have shown that these analytical calculations work well for estimating the jitter when comparisons are made with simulation results under different recording conditions and media variations. One of the simulations showed that the grain pitch has a greater effect on the transition jitter than on the read width. Pituso et al. [13,14] have simulated the magnetic recording process in a two-dimensional (2-D) format. The simulation demonstrates magnetic footprints of HAMR technology where heating is based on the relationship of magnetic properties with temperature. The behavior of magnetic properties with temperature is used to identify the hotspot for the simulation. Hernandez et al. [15] proposed parameters that can achieve the high areal density in HAMR technology.

From many studies [10,11,12,19,21,22], the transition jitter can be obtained by the standard deviation from a read-back signal at the zero-crossing position. In this paper, the transition jitter was shown in another form of the transition jitter by indicating the position of the transition bits in a 2-D format. Since the magnetic footprint simulation was analyzed for the transition jitter simulation in a 2-D format, this simulation was analyzed to resemble the magnetic footprint experimental analysis imaging shown in the 2-D format of spin-stand microscopy. The spin-stand is a machine that can characterize of the magnetic footprints for analysis, such as transition curvature analysis [23,24,25]. Therefore, this paper aimed to maintain a reasonable level of performance from increasing both the linear density and the track density. We also proposed that the magnetic footprint simulation was simulated for the transition jitter simulation in a 2-D format. The *L*1_0_-FePt magnetic material’s properties depend on the temperature used to identify the hotspot area for the heating simulation in the Voronoi medium. The micromagnetic modeling is based on the Landau–Lifshitz–Gilbert (LLG) equation. The lowest transition jitter average simulation was investigated at the areal density of 4.1 Tb/in^2^. In addition, the lowest transition jitter average was investigated at ultrahigh areal densities from 4.1 to 8.9 Tb/in^2^ in HAMR technology.

## 2. Materials and Methods

In this work, the micromagnetic simulation was based on the LLG equation, as shown in Equation (1) [13,26,27]:

(1) $$\frac{d\vec{M}}{dt}=-\gamma \vec{M}\times {\vec{H}}_{eff}-\frac{\gamma \alpha }{M_{s}}\vec{M}\times \left(\vec{M}\times {\vec{H}}_{\mathrm{eff}}\right)$$

where $\vec{M}$ is the magnetization vector, γ is the gyromagnetic ratio, α is the damping constant, and *M*_s_ is the saturation magnetization. The effective field, ${\vec{H}}_{\mathrm{eff}}$, includes the exchange, demagnetizing, anisotropy, and Zeeman fields. The simulation was implemented by the object-oriented micromagnetic framework (OOMMF) software [28].

The magnetic recording simulation process of HAMR technology used the correlation between the temperature and the properties of the magnetic materials to create the hotspot area. Therefore, the hotspot area model used the Brillouin function equation, as shown in Equations (2)–(4) [13,14]:

(2) $$M_{s}\left(T\right)=M_{s}\left(0\right)\left[\frac{2J+1}{2J}\coth\left(\frac{2J+1}{2J}\beta \right)-\frac{1}{2J}\coth\left(\frac{\beta }{2J}\right)\;\right]$$

and

(3) $$\frac{K_{u}\left(T\right)}{K_{u}\left(0\right)}={\left(\frac{M_{s}\left(T\right)}{M_{s}\left(0\right)}\right)}^{n}$$

where

(4) $$\beta =3\left(1-\frac{T}{T_{C}}\right)$$

where *J* is the total angular momentum quantum number, *n* is a medium film series factor, *T* is the temperature, *T*_C_ is the Curie temperature, *H*_k_ is the anisotropy field, and *K*_u_ is the magnetocrystalline anisotropy constant.

**Figure 1:**
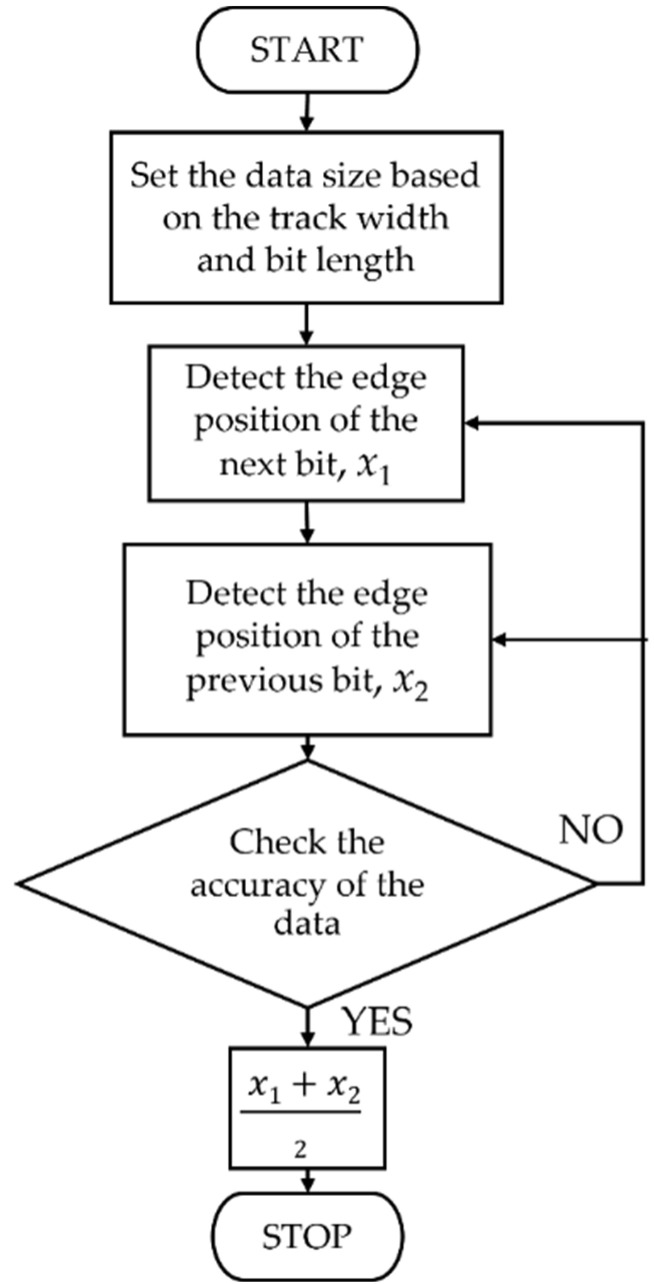
The zig-zag boundary procedure flow chart.

The shape of the hotspot area was a squircle, the shape of the applied field was rectangular, and they were the same width. The writing model was five tracks in a medium of each bit length, and a single-tone sequence was written on a track of 31 bits and 30 boundaries. The Voronoi grain medium had dimensions of 1000 nm × 1500 nm and a thickness of 6 nm. The medium model had a resolution of 0.25 pixels per 1 nm in the x–y plane. The average grain size was 5.1 nm [15] with the standard deviation of 0.08 nm, and the grain boundary width was about 1–2 nm. The mesh cell size of the micromagnetic simulation in the x–y plane was 1 nm × 1 nm, and in the *Z*-axis it was 3 nm. The magnetic properties at room temperature of the *L*1_0_-FePt medium were as follows: *M*_s_ (300 K) = 1.100 MA/m and *K*_u_ (300 K) = 7 MJ/m^3^. The *T* for heating in the HAMR process was 700 K, and the *T*_C_ was 710 K. The write head field was 10 kOe along the z-direction, and *J* was 0.85 at a medium film series factor, *n*, of 2.15 for the *L*1_0_-FePt magnetic material [14,15]. The intragrain exchange stiffness constant was 12 pJ/m, and the intergrain exchange stiffness was 0 J/m [13]. MATLAB [29] was used for the Voronoi medium modeling and the $\sigma_{jitter}$ that could be obtained from the zig-zag boundary procedure flow chart for the transition jitter simulation, as shown in Figure 1.

The $\sigma_{jitter}$ was the standard deviation of each zero-crossing position, as shown in Equation (5) [10,11,12,19,21,22]:

(5) $$\sigma_{jitter}=\sqrt{\frac{1}{N-1}\sum_{i=1}^{N}{\left(x_{i}-{\bar{x}}_{m}\right)}^{2}}$$

where $x_{i}$ is zero-crossing position, ${\bar{x}}_{m}$ is an average position, and *N* is a total number of transitions.

The transition jitter average, ${\bar{\sigma }}_{jitter}$, was calculated by the summation of $\sigma_{jitter}$ of each track in a medium divided by the total number of tracks, *N_t_*, as shown in Equation (6).

(6) $${\bar{\sigma }}_{jitter}=\frac{1}{N_{t}}\sum_{i=1}^{N_{t}}\sigma_{jitter}{}_{i}$$

### 2.1. Minimum Transition Jitter at the Areal Density of 4.1 Tb/in^2^

The simulation parameters were determined under the scope of the AD at 4.1 Tb/in^2^ for finding the lowest ${\bar{\sigma }}_{jitter}$, as shown in Table 1.

### 2.2. Transition Jitter at Ultrahigh Areal Density of 4.1–8.9 Tb/in^2^

In Table 2, the bit length of 9 nm was selected to investigate the ${\bar{\sigma }}_{jitter}$ at ultrahigh areal densities from 4.1 to 8.9 Tb/in^2^ by decreasing the track width.

## 3. Results and Discussions

### 3.1. Minimum Transition Jitter at the Areal Density of 4.1 Tb/in^2^

**Figure 2:**
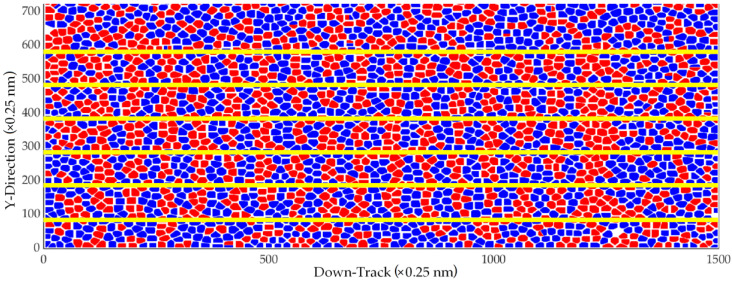
Magnetic footprints of 9 nm bit lengths with a track width of 16.5 nm.

**Figure 3:**
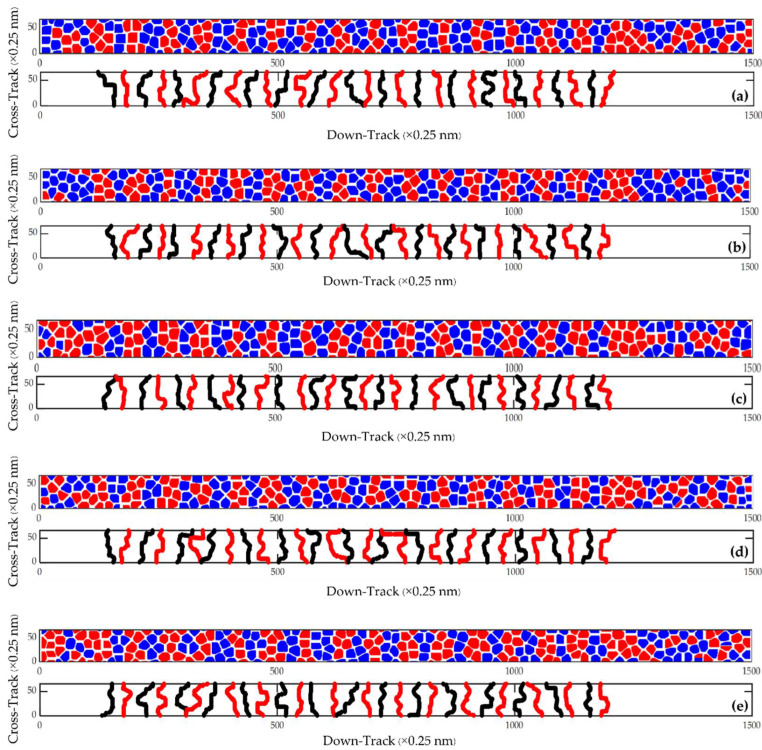
The magnetic footprints (**upper**) at a bit length of 9 nm with a track width of 16.5 nm and the boundary between bits (**lower**) of the (**a**) 1st, (**b**) 2nd, (**c**) 3rd, (**d**) 4th, and (**e**) 5th tracks.

Figure 2 shows the magnetic footprint simulation result of 31 bits per track (between the yellow lines) at the areal density of 4.1 Tb/in^2^ (9 nm bit length and 16.5 nm track width). The magnetic footprints of each track were analyzed to show the transition boundaries in a 2-D format, as shown in Figure 3.

**Figure 4:**
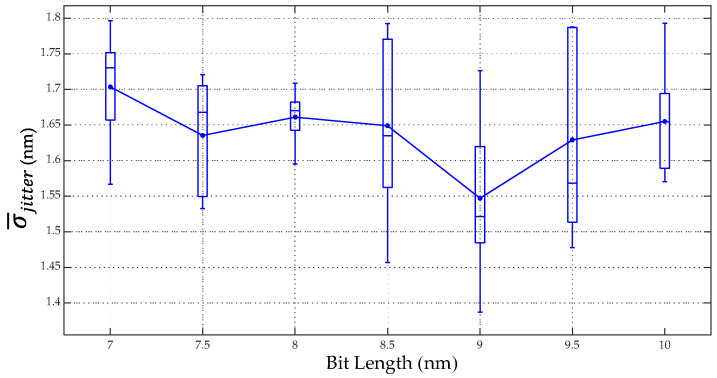
Relationship between the ${\bar{\sigma }}_{jitter}$ and bit length.

Figure 4 shows the results of the ${\bar{\sigma }}_{jitter}$ at the areal density of 4.1 Tb/in^2^. It was found that the ${\bar{\sigma }}_{jitter}$ values of bit lengths of 7, 7.5, 8, 8.5, 9, 9.5, and 10 nm were 1.703, 1.635, 1.661, 1.649, 1.547, 1.629, and 1.655 nm, respectively. The lowest ${\bar{\sigma }}_{jitter}$ was 1.547 nm at a bit length of 9 nm and a track width of 16.5 nm. The results in Figure 4 also show that the fluctuations in the ${\bar{\sigma }}_{jitter}$ at bit lengths of 7 to 10 nm are probably from the grain shape or the grain size distribution. The ${\bar{\sigma }}_{jitter}$ of the 7 nm bit length increased when the reducing bit length approached the grain size because the bit length of 6 nm cannot be simulated. The results of the micromagnetic simulations also found that some of the bits did not have the magnetization switching in the grain because some parts of the grain were not in the hotspot area. Therefore, the broad zig-zag boundary was the effect of reducing bit length approaches to the grain size.

### 3.2. Transition Jitter at Ultrahigh Areal Densities of 4.1–8.9 Tb/in^2^

**Figure 5:**
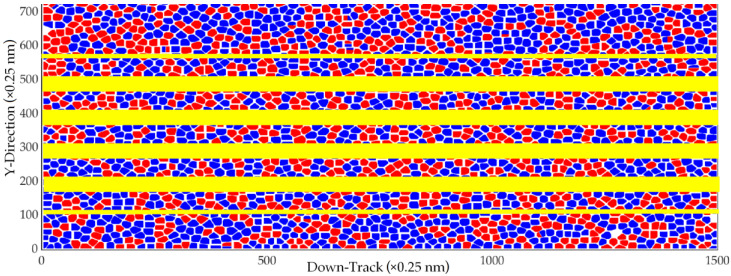
Magnetic footprints at AD of 8.9 Tb/in^2^.

**Figure 6:**
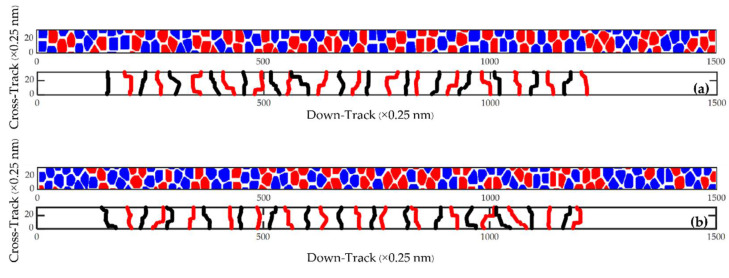

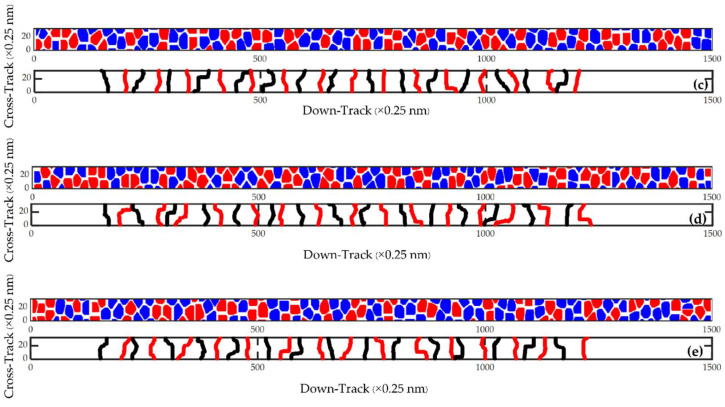
The magnetic footprints (**upper**) at a bit length of 9 nm with a track width 8 nm and the boundary between bits (**lower**) of the (**a**) 1st, (**b**) 2nd, (**c**) 3rd, (**d**) 4th, and (**e**) 5th tracks.

Figure 5 shows the magnetic footprint simulation result that was investigated from the areal density of 4.1 Tb/in^2^, and this is the magnetic footprint simulation result of 31 bits per track (between the yellow lines) at the ultrahigh areal densities from 4.1 to 8.9 Tb/in^2^. The magnetic footprints of each track in Figure 5 were analyzed to show the transition boundaries in a 2-D format, as shown in Figure 6.

**Figure 7:**
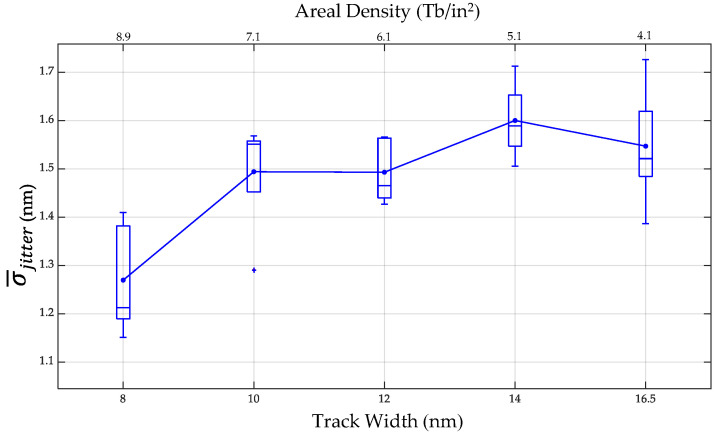
The ${\bar{\sigma }}_{jitter}$ for track width and areal density variation.

The results in Section 3.1 show that the bit length of 9 nm had the lowest ${\bar{\sigma }}_{jitter}$. In this section, the track width of the 9 nm bit length was selected to investigate the ${\bar{\sigma }}_{jitter}$ at ultrahigh areal densities from 4.1 to 8.9 Tb/in^2^, and Figure 7 shows the ${\bar{\sigma }}_{jitter}$ for track width and areal density variation. It was found that the ${\bar{\sigma }}_{jitter}$ values of each track width at 9 nm bit lengths of 8, 10, 12, 14, and 16.5 nm were 1.270, 1.490, 1.493, 1.60, and 1.547 nm, respectively. The lowest ${\bar{\sigma }}_{jitter}$ was 1.270 nm at an 8 nm track width. The trend of ${\bar{\sigma }}_{jitter}$ reduced with increases in the areal density from 4.1 to 8.9 Tb/in^2^ (or decreasing track width from 16.5 to 8 nm.). The trend of ${\bar{\sigma }}_{jitter}$ reduced with decreasing track width. It was likely due to the zero-crossing position being outside the track area, and this trend is consistent with those in the literature [12,30].

## 4. Conclusions

In this paper, we present the transition jitter simulation in the 2-D format of HAMR technology at ultrahigh areal densities from 4.1 to 8.9 Tb/in^2^. The *L*1_0_-FePt magnetic material was used as the magnetic medium for future magnetic recording. The OOMMF was used for recording process simulation, and the MATLAB program was used to simulate the transition jitter in a 2-D format. The areal density of 4.1 Tb/in^2^ has the lowest ${\bar{\sigma }}_{jitter}$ of 1.547 nm (9 nm bit length and 16.5 nm track width). The areal densities from 4.1 to 8.9 Tb/in^2^ had the lowest ${\bar{\sigma }}_{jitter}$ of 1.270 nm (9 nm bit length and 8 nm track width). These results can be the guidelines for future magnetic recording technology development.

## Figures and Tables

**Table 1:** Bit length, track width, and hotspot size use in the simulation at the areal density of 4.1 Tb/in^2^.

Areal Density (Tb/in^2^)	Bit Length (nm)	Track Width (nm)	Hotspot Size (nm)
4.1 [15]	7 [15]	22.5 [15]	28
	7.5	21	26.5
	8 [15]	19.5 [15]	25 [15]
	8.5	18	23.5
	9	16.5	22
	9.5	15	20.5
	10	13.5	19

**Table 2:** Track width and hotspot size at ultrahigh areal density from 4.1 to 8.9 Tb/in^2^.

Bit Length (nm)	Track Width (nm)	Hotspot Size (nm)	Areal Density (Tb/in^2^)
9	16.5	22	4.1
	14	19.5	5.1
	12	17.5	6.1
	10	15.5	7.1
	8	13.5	8.9

## Data Availability

Not applicable.
